# Longitudinal effects of aging on plasma proteins levels in older adults – associations with kidney function and hemoglobin levels

**DOI:** 10.1371/journal.pone.0212060

**Published:** 2019-02-25

**Authors:** Lars Lind, Johan Sundström, Anders Larsson, Erik Lampa, Johan Ärnlöv, Erik Ingelsson

**Affiliations:** 1 Department of Medical Sciences, Uppsala University, Uppsala, Sweden; 2 Uppsala Clinical Research Center, Uppsala, Sweden; 3 School of Health and Social Studies, Dalarna University, Falun, Sweden; 4 Division of Family Medicine, Department of Neurobiology, Care Sciences and Society, Karolinska Institutet, Huddinge, Sweden; 5 Department of Medical Sciences, Molecular Epidemiology and Science for Life Laboratory, Uppsala University, Uppsala, Sweden; 6 Department of Medicine, Division of Cardiovascular Medicine, Stanford University School of Medicine, Stanford, CA, United States of America; 7 Stanford Cardiovascular Institute, Stanford University, Stanford, CA, United States of America; 8 Stanford Diabetes Research Center, Stanford University, Stanford, CA, United States of America; International University of Health and Welfare, School of Medicine, JAPAN

## Abstract

**Background:**

A targeted proteomics chip has been shown to be useful to discover novel associations of proteins with cardiovascular disease. We investigated how these proteins change with aging, and whether this change is related to a decline in kidney function, or to a change in hemoglobin levels.

**Material and methods:**

In the Prospective Investigation of the Vasculature in Uppsala Seniors (PIVUS) study, including 1,016 participants from the general population aged 70 at baseline, 84 proteins were measured at ages 70, 75, 80. At these occasions, glomerular filtration rate (eGFR) was estimated and the hemoglobin levels were measured.

**Results:**

Sixty-one of the 84 evaluated proteins changed significantly during the 10-year follow-up (multiple testing-adjusted alpha = 0.00059), most showing an increase. The change in eGFR was inversely related to changes of protein levels for the vast majority of proteins (74%). The change in hemoglobin was significantly related to the change in 40% of the evaluated proteins, with no obvious preference of the direction of these relationships.

**Conclusion:**

The majority of evaluated proteins increased with aging in adults. Therefore, normal ranges for proteins might be given in age-strata. The increase in protein levels was associated with the degree of reduction in eGFR for the majority of proteins, while no clear pattern was seen for the relationships between the proteins and the change in hemoglobin levels. Studies on changes in urinary proteins are warranted to understand the association between the reduction in eGFR and increase in plasma protein levels.

## Introduction

It is known that circulating levels of certain proteins, like C-reactive protein (CRP), cardiac troponin I, growth-differentiation factor-15 (GDF-15) and N-terminal pro b-type natriuretic peptide (NT-proBNP), increase with aging [1]. However, few studies have attempted to investigate if this is a common theme for a larger number of proteins. In one study using untargeted proteomics in a cross-sectional sample, it was shown that thirteen proteins out of more than one thousand measured were related to age following adjustment for multiple testing [2]. Also, other studies have shown relationships between the proteome and age [3–5], but as far as we are aware, no prior studies have addressed associations of multiple proteins to aging in a longitudinal fashion, with repeated protein measurements in the same individuals over a longer time period in adults. Longitudinal studies of changes in the proteome exist in pediatric populations [6, 7], but this part of life is characterized by growth and the change in the proteome would presumably reflect other processes than seen in adult life, especially among elderly.

The kidney is a key regulator of circulating protein levels, since many proteins are excreted with the urine. Patients with advanced chronic renal failure show elevated levels of many proteins in plasma [8]. It has also been shown that numerous protein biomarkers increase with declining glomerular filtration rate (GFR) in a cross-sectional study [9]. Furthermore, in the most extensive cross-sectional testing of the proteome in relation to measured GFR published so far, many proteins were found to be negatively related to GFR and an increase in protein levels was more common than a decline when individuals with low GFR was compared to those with a normal GFR [10]. Based on these previous findings, we hypothesized that the longitudinal changes in protein levels during ten years of follow-up in our sample would be associated with changes in eGFR.

Another general factor that could govern protein levels is the amount of plasma water. During hemoconcentration, plasma concentrations of proteins, including hemoglobin levels, will increase [11]. Therefore, we also hypothesized that the longitudinal changes in protein levels between age 70 and 80 would be associated with changes in hemoglobin levels.

Using the proximal extension assay (PEA) technique for measurement of multiple proteins on a targeted proteomics chip, we have recently discovered new proteins to be related to atherosclerosis and cardiovascular disease in the Prospective Investigation of the Vasculature in Uppsala Seniors (PIVUS) study [12–14]. To gain further insights into how these proteins change with aging, and how this is linked to changes in kidney function and hemoglobin levels, we performed measurements using the protein array at ages 70, 75 and 80 in the PIVUS cohort. Our primary aim was to evaluate changes in circulating protein levels over 10 years in this cohort. Secondly, we wanted to investigate if these changes were associated with changes in eGFR and hemoglobin levels seen during the same period.

## Materials and methods

### Sample

The population-based Prospective Investigation of the Vasculature in Uppsala Seniors (PIVUS) study was initiated in 2001 to collect data on multiple measures of cardiovascular function. At the baseline investigation in 2001–2004, 1,016 subjects all aged 70 years were enrolled (50% women). Follow-up investigations have been performed after five (n = 826) and ten years (n = 602). A detailed description of the cohort is available online (www.medsci.uu.se/pivus) and in prior publications [15].

Basic characteristics of the sample are given in Table A in S1 File.

### Measurements

The participants were investigated in the morning after an overnight fast. Except for measurements of traditional lipids and fasting glucose–that were measured at the Clinical chemistry department in fresh samples–plasma was stored in a freezer (-80°C) for later analyses. A history of diseases and drug use was collected.

Plasma creatinine and cystatin C were measured by a standard enzymatic method and by an enhanced turbidimetric method, respectively, and a validated formula to estimate eGFR using both of these markers was used [16]. Hemoglobin levels were analyzed using a CELL-DYN Sapphire Hematology System (Abbott Laboratories, Abbott Park, IL, US) and reported in g/L.

Proteins were measured using the proximal extension assay (PEA) technique [17] on a commercial proteomics array with 92 preselected proteins known/suggested to be involved in cardiovascular disease (Olink Proseek Multiplex CVD I^96×96^, Olink, Uppsala, Sweden). The array includes 92 oligonucleotide-labeled antibody probe pairs that are allowed to bind to their respective target present in the sample. The PEA technique has a major advantage over conventional multiplex immunoassays in that only correctly matched antibody pairs give rise to a signal, yielding an exceptionally high specificity. PEA is a homogeneous assay that uses pairs of antibodies equipped with DNA reporter molecules. When binding to their correct targets, they give rise to new DNA amplicons each ID-barcoding their respective antigens. The amplicons are subsequently quantified using a Fluidigm BioMark HD real-time PCR platform. Data is normalized and transformed using internal extension controls and inter-plate controls, to adjust for intra- and inter-run variation as described earlier [17]. The Proseek Multiplex CVD I^96×96^ has been shown to have high reproducibility and repeatability with mean intra-assay and inter-assay coefficients of variation of 8% and 12%, respectively; and the average inter-site variation has been reported to be 15% [17]. The final assay readout is given in Normalized Protein eXpression (NPX), which is an arbitrary unit on log_2_-scale where a high value corresponds to a higher protein expression. All measurements were performed in the same fashion at all three occasions. Of the 92 proteins, 84 showed a call rate >75% at all three time points, and these proteins were included in further analyses. Details regarding the protein arrays, quality control and processing of data in the PIVUS study has been published previously [13, 14].

### Statistical analysis

The distributions of all protein levels were skewed to the right and were therefore log_2_-transformed to achieve a normal distribution. In the final analysis, the log_2_-transformed values for the proteins were transformed to a standard deviation (SD)-scale, so the regression coefficient (beta) were comparable between the proteins.

#### Changes in protein levels during aging

First, the change in the 84 proteins over 10 years (three measurements) were evaluated by mixed random effect models with the proteins (one protein in each model) as dependent variable, and time as the independent variable and sex as confounder (age same in all subjects). Thus, the random effect is the individual and the fixed effects are time and sex. This general model is: protein = time sex (random effect for individual).

This primary analysis included all available data from all subjects at all time-points. The mixed model procedure automatically takes into account the effect of missing data, but as a sensitivity analysis we also performed the analysis in the 571 subjects with data at all three time-points.

To evaluate if the changes in proteins were different in males and females, the models were also run including an interaction term between time and sex.

To evaluate if hemoglobin (as a measure of hemoconcentration) affects the change in proteins over time, we as a secondary analysis also included hemoglobin as a co-variate in the models (a fixed effect).

The relationship between the molecular weight and the beta-value for change in protein levels over time was assessed by Spearman rank correlation.

To test if the change over time for the proteins were influenced to a major degree by disorders or medication in the sample, a sensitivity analysis was performed in the 303 individuals free from any medication using the same mixed model as described above. The beta-values for all 84 proteins in that analysis were related to the beta-values obtained in the total sample be Pearson´s correlation coefficient.

#### Correlated protein changes

The pairwise Spearman rank correlations between ten-year absolute changes in protein levels were visualized in a circular plot. The circular plot depicts proteins on the edge of a circle with lines connecting highly correlated (rank correlation > 0.6) proteins. While the mixed models use data from all three time-points, this visualization can only use the 70 and 80-year data.

#### Association vs the change in eGFR

First, the change in eGFR over 10 years (three measurements) were evaluated by mixed random effect models with eGFR as dependent variable, and time as the independent variable and sex as confounder (age same in all subjects). Thus, the random effect is the individual and the fixed effects are time and sex. This general model is: eGFR = time sex (random effect for individual).

Thereafter, the relationships between changes over 10 years in the levels of the 84 proteins and the changes over 10 years in eGFR were examined. Also, for this purpose mixed random effect models were used, and eGFR used as an independent variable. One model was analyzed for each protein. The independent variable was split into a between-individual component, which is the first observation for the individual, and a within-individual component, which is the difference between the measurements at future time points and the first measurement. Thus, the between-individual component is related to the mean of the three measurements of the proteins, while the between-individual component, as a single term, relates the change in each protein to the change in eGFR. The theory and assumptions behind this model, as well as the detailed formula is provided in the “Longitudinal and cross-sectional information” paragraph of the chapter “Some aspects of the design of longitudinal studies" in [18]. The general formula is; Y_ij_ = Z_i_beta_0_ –X_i1_beta_C_ + (X_ij_-X_i1_)beta_L +_ e_ij_, where Y is a protein, X is eGFR, i is the individual, j the time, beta_C_ is the coefficient for the first observation and beta_L_ is the coefficient for change over time. Confounders and the intercept are given as Z_i_beta_0._

Thus, baseline GFR (at age 70), the change in GFR and sex are the fixed effect and individual is the random effect. The general formula is: protein = eGFR_change eGFR_baseline sex (random effect for individual). We used only a random intercept, since inclusion of also a random slope made the models not to converge in most cases. We used the default correlation matrix of the command “mixed”, which is independent, and works well in cases with a random intercept.

#### Association vs the change in hemoglobin

First, the change in hemoglobin over 10 years (three measurements) were evaluated by mixed random effect models with hemoglobin as dependent variable, and time as the independent variable and sex as confounder (age same in all subjects). Thus, the random effect is the individual and the fixed effects are time and sex. This general model is: hemoglobin = time sex (random effect for individual).

Thereafter, the relationships between changes over 10 years in the levels of the 84 proteins and the changes over 10 years in hemoglobin levels were examined in a similar fashion as the change in eGFR.

#### Combined association of changes in eGFR and hemoglobin level

We estimated the relationships between changes over 10 years in the levels of the 84 proteins and the changes over 10 years in both eGFR and hemoglobin levels in a similar fashion using the general formula; protein = eGFR_change eGFR_baseline hemoglobin_change hemoglobin_ baseline sex (random effect for individual). In this case, changes in both eGFR and hemoglobin as well as baseline values of both eGFR and hemoglobin were together with sex the fixed effects.

#### Significance level

We regarded a Bonferroni-corrected p-value of 0.00059 (0.05/84) as significant in each of the analyses. Since many of the proteins are highly correlated, we did not adjust for analyzing two outcomes (eGFR and hemoglobin), to strike a reasonable balance between risk for false negatives and false positives.

STATA14 was used for analysis (Stata Inc, College Station, TX, USA).

## Results

### Changes in protein levels during aging

**Fig 1:**
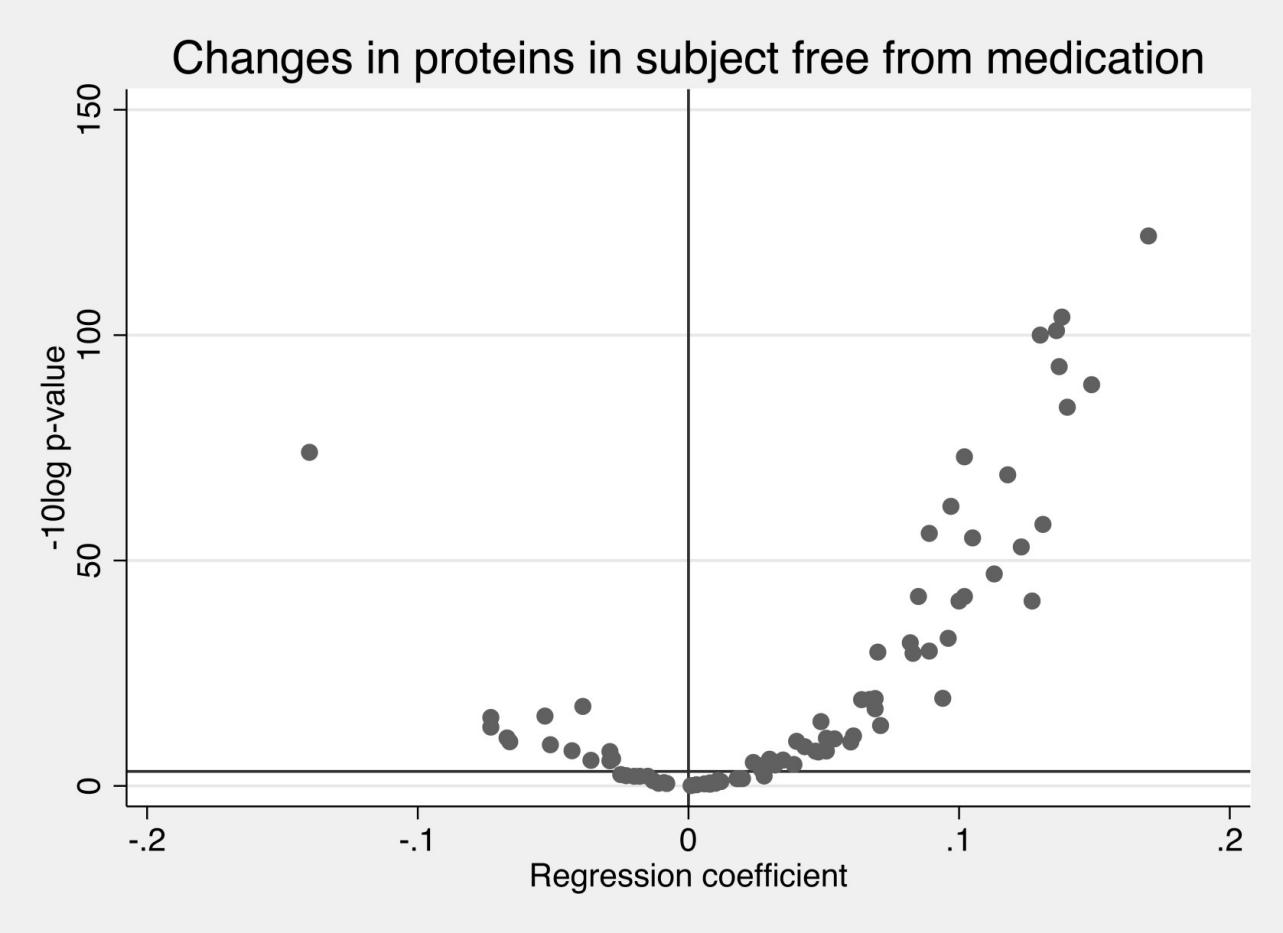
Effect sizes (regression coefficients) of changes of 84 proteins over time and the–log_10_ p-value for corresponding changes. The Bonferroni-corrected p-value is shown as the horizontal line. The regression coefficient (beta) gives the change per year on a SD-scale.

In analyses of the total sample, 62 of the 84 evaluated proteins changed significantly over time, most showing an increase. (Bonferroni-adjusted alpha<0.00059, Fig 1 and Table 1).

**Table 1:** Change in 84 proteins over 10-year follow-up with measurements at ages 70, 75 and 80 years.

Protein	Beta	SE	p-value
Adrenomedullin (AM)	.170	.007	1.0e-122
Urokinase plasminogen activator surface receptor (U-PAR)	.138	.006	1.0e-104
Osteoprotegerin (OPG)	.136	.006	2.1e-101
Interleukin-27 subunit alpha (IL27-A)	.130	.006	8.0e-100
N-terminal pro-B-type natriuretic peptide (NT-pro-BNP)	.137	.007	4.5e-93
Tissue-type plasminogen activator (t-PA)	.149	.007	7.9e-89
Proteinase-activated receptor 1 (PAR-1)	.140	.007	1.4e-84
Caspase-8 (CASP-8)	-.140	.008	1.9e-74
Growth/differentiation factor 15 (GDF-15)	.102	.006	1.0e-73
Endothelial cell-specific molecule 1 (ESM-1)	.118	.007	2.6e-69
Fibroblast growth factor 23 (FGF-23)	.097	.006	2.6e-62
Pentraxin-related protein PTX3 (PTX3)	.131	.008	2.2e-58
Matrix metalloproteinase-12 (MMP-12)	.089	.006	2.7e-56
Vascular endothelial growth factor D (VEGF-D)	.105	.007	2.0e-55
Spondin-1 (SPON1)	.123	.008	1.5e-53
Agouti-related protein (AGRP)	.113	.008	1.3e-47
Fractalkine (CX3CL1)	.102	.007	1.2e-42
CD40L receptor (CD40)	.085	.006	1.8e-42
Proto-oncogene tyrosine-protein kinase Src (SRC)	.127	.009	1.6e-41
TNF-related apoptosis-inducing ligand (TRAIL)	.102	.007	9.5e-41
Heparin-binding EGF-like growth factor (HB-EGF)	.096	.008	1.89e-33
Hepatocyte growth factor (HGF)	.082	.007	1.86e-32
Kallikrein-6 (KLK6)	.089	.008	1.28e-30
ST2 protein (ST2)	.070	.006	2.21e-30
Interleukin-16 (IL-16)	.083	.007	4.05e-30
P-selectin glycoprotein ligand 1 (PSGL-1)	.094	.01	3.83e-20
Cystatin-B (CSTB)	.069	.007	4.33e-20
Vascular endothelial growth factor A (VEGF-A)	.067	.007	6.80e-20
Pappalysin-1 (PAPPA)	.064	.007	7.54e-20
Membrane-bound aminopeptidase P (mAmP)	-.039	.004	2.41e-18
Macrophage colony-stimulating factor 1 (CSF-1)	.069	.008	8.27e-18
Matrix metalloproteinase-1 (MMP-1)	-.053	.007	3.34e-16
Myeloperoxidase (MPO)	-.073	.009	6.92e-16
TNF-related apoptosis-inducing ligand receptor 2 (TRAIL-R2)	.049	.006	5.69e-15
Lectin-like oxidized LDL receptor 1 (LOX-1)	.071	.009	4.20e-14
Epidermal growth factor (EGF)	-.073	.01	9.41e-14
Follistatin (FS)	.061	.009	8.49e-12
Protein S100-A12 (EN-RAGE)	-.067	.01	2.43e-11
Eosinophil cationic protein (ECP)	.051	.008	2.84e-11
Interleukin-6 (IL-6)	.054	.008	3.79e-11
Receptor for advanced glycosylation end products (RAGE)	.040	.006	1.34e-10
Cathepsin L1 (CTSL1)	-.066	.01	1.66e-10
C-C motif chemokine 20 (CCL20)	.060	.009	1.85e-10
TNF-related activation-induced cytokine (TRANCE)	-.051	.008	7.73e-10
Placenta growth factor (PlGF)	.043	.007	2.09e-09
Platelet-derived growth factor subunit B (PDGF subunit B)	-.043	.008	1.65e-08
Kallikrein-11 (hK11)	.051	.009	1.88e-08
Tissue factor (TF)	.047	.008	2.04e-08
Leptin (LEP)	-.029	.005	2.49e-08
Monocyte chemotactic protein 1 (MCP-1)	.048	.009	3.09e-08
Fatty acid-binding protein 4 (FABP4)	-.028	.006	9.94e-07
Interleukin-6 receptor subunit alpha (IL-6RA)	.030	.006	1.25e-06
Chitinase-3-like protein 1 (CHI3L1)	.035	.007	1.93e-06
C-X-C motif chemokine 6 (CXCL6)	-.036	.008	2.20e-06
Matrix metalloproteinase-3 (MMP-3)	-.029	.006	2.78e-06
Galectin-3 (Gal-3)	.031	.007	3.28e-06
Tumor necrosis factor receptor 2 (TNF-R2)	.030	.007	3.63e-06
T-cell immunoglobulin and mucin domain 1 (TIM-1)	.024	.005	6.52e-06
Tumor necrosis factor receptor 1 (TNF-R1)	.029	.007	9.51e-06
Tumor necrosis factor ligand superfamily member 14 (TNFSF14)	.039	.009	.0000183
Platelet endothelial cell adhesion molecule (PECAM-1)	.032	.008	.0000241
C-C motif chemokine 4 (CCL4)	.027	.007	.0002472
Interleukin-1 receptor antagonist protein (IL-1RA)	-.025	.008	.0031852
Angiopoietin-1 receptor (TIE2)	-.023	.008	.0054946
Growth hormone (GH)	.028	.01	.0065182
E-selectin (SELE)	-.015	.006	.0075581
Thrombomodulin (TM)	-.018	.007	.0076076
Resistin (RETN)	-.020	.008	.0077487
C-X-C motif chemokine 16 (CXCL16)	.020	.009	.0252256
Tumor necrosis factor receptor superfamily member 6 (FAS)	.018	.008	.0260263
Myoglobin (MB)	.019	.008	.0261793
Galanin peptides (GAL)	.011	.006	.0564566
C-X-C motif chemokine 1 (CXCL1)	-.013	.007	.0733593
C-C motif chemokine 3 (CCL3)	.012	.007	.1115752
Prolactin (PRL)	.010	.007	.1855251
Interleukin-18 (IL-18)	-.009	.007	.1974891
Cathepsin D (CTSD)	.008	.007	.2256375
Interleukin-8 (IL-8)	-.011	.009	.2643662
Matrix metalloproteinase-10 (MMP-10)	.010	.009	.267806
Dickkopf-related protein 1 (DKK-1)	-.008	.007	.3007548
Ovarian cancer-related tumor marker CA 125 (CA-125)	.006	.006	.367675
Heat shock 27 kDa protein (HSP 27)	.008	.01	.4298231
Renin (REN)	.003	.005	.5746617
Stem cell factor (SCF)	.001	.006	.8936469

The betas show the direction and magnitude of the change per year on a SD scale (to increase cross-comparability). *P*< 0.00059 is regarded as significant (Bonferroni-correction for 84 proteins).

**Fig 2:**
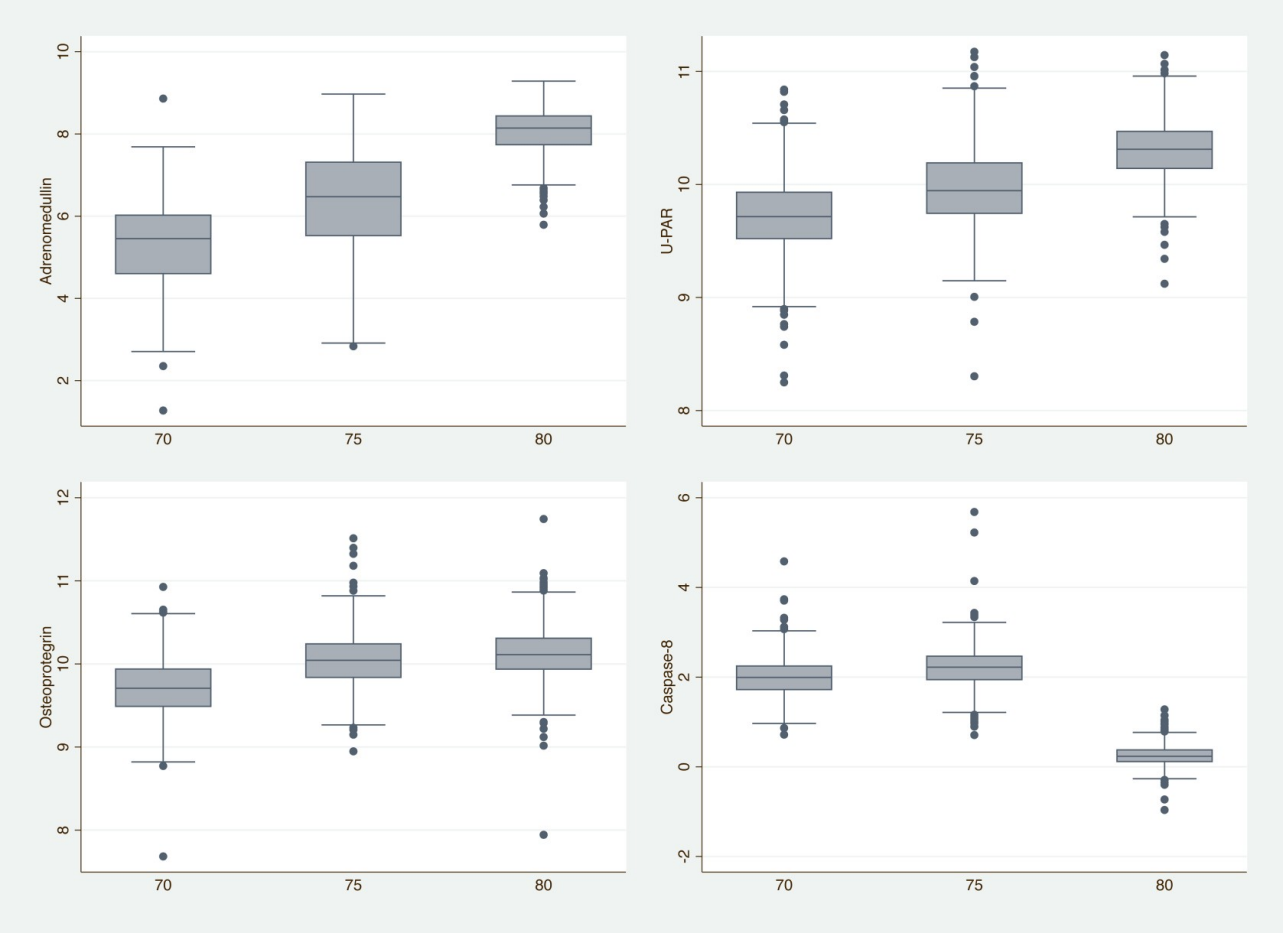
Box plot showing the Normalized Protein eXpression (NPX) values for the 3 proteins showing the most significant increments over time and the protein showing the most significant decline over time. U-PAR = Urokinase plasminogen activator surface receptor.

Adrenomedullin (AM), Urokinase plasminogen activator surface receptor (U-PAR), Osteoprotegerin (OPG), Interleukin-27 subunit alpha (IL27-A), and N-terminal pro-B-type natriuretic peptide (NT-pro-BNP) were the five proteins that increased the most over the 10-year period. As an illustration, levels of the 3 proteins showing the largest increase, and the top protein showing a decline are shown in Fig 2.

Very similar results were obtained when only data from the 571 subjects with data from all three timepoints were used in the analysis. In this case, 74 of the proteins changed significantly with a similar enrichment for protein increases over time.

A significant interaction between time and sex was found for 12 of the proteins (Table B in S1 File). For 10 of those proteins, the changes over 10 years were more pronounced in men than in women.

A very similar result as described above was obtained if the hemoglobin level was included in the model as a co-variate (to adjust for hemoconcentration). In that case, 58 proteins increased significantly over time, while 13 declined significantly over time.

When the theoretical molecular weights of the different proteins were calculated, no significant relationship was seen the molecular weight and the change over time in protein levels (p = 0.17).

We also repeated the analyses in 303 participants free from all prescription medication at all three time-points in a sensitivity analysis. In these analyses, 61 of the 84 evaluated proteins changed significantly over time (Bonferroni-adjusted alpha<0.00059). The correlation of regression coefficients from the main analysis and this sensitivity analysis was 0.98. For this reason, and to maximize statistical power, the full sample was used for all subsequent analyses.

### Correlated protein changes

As seen in a circle plot of highly correlated protein changes (Figure A in S1 File), many protein levels tracked over the evaluated 10-year period. Judging by the large number of correlated changes, CD40 ligand (CD40), Cystatin-B (CSTB), Tumor Necrosis Factor receptors 1 and 2 (TNF-R1 and R2), Thrombomodulin (TM), Interleukin-6 receptor subunit alpha (IL-6RA) and C-C motif chemokine 3 (CCL3) seemed like nodes that were related to the changes of five or more other proteins.

### Association vs the change in eGFR

**Fig 3:**
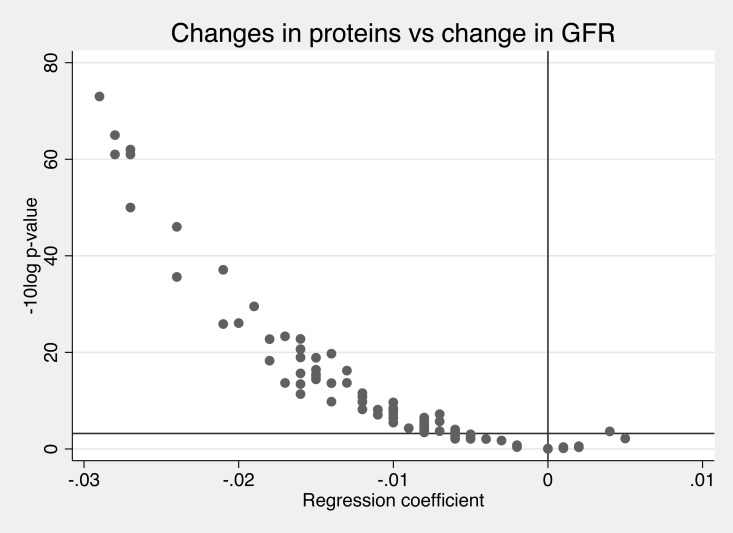
Associations between the change in kidney function (glomerular filtration rate, GFR) and change in 84 prteins over time (regression coefficient) and the–log_10_ p-value for these associations. The Bonferroni-corrected p-value is shown as the horizontal line. The regression coefficient (beta) gives the relationship between the change in protein levels (on a SD-scale) vs the change in GFR (in mL/min/1.73m2).

eGFR declined significantly during the 10 years (p<e-100). The change in eGFR was inversely related to the change in the majority of the evaluated proteins (74%) (Fig 3). The top five inverse relationships between change in GFR and change in protein levels were seen for Cystatin-B (CSTB), Tumor necrosis factor receptor 1 (TNF-R1), CD40L receptor (CD40), Tumor necrosis factor receptor 2 (TNF-R2), TNF-related apoptosis-inducing ligand receptor 2 (TRAIL-R2).

### Association vs the change in hemoglobin

At age 70, the hemoglobin level was 138 (SD 11) and declined to 136 (SD 11) g/L after 10 years (p = 5.3e-04). The change in hemoglobin was significantly related to the change in 40% of the evaluated proteins (Figure B in S1 File). As could be seen in the figure, there was no obvious preference of the direction of these relationships.

### Combined association of changes in eGFR and hemoglobin level

A positive relationship existed between the change in eGFR and the change in hemoglobin (beta 0.10, SE 0.03, Pearson´s correlation coefficient 0.11, p-value = 7.9e-04).

Therefore, we performed an analysis with the proteins as dependent variables (in separate models for each protein) and both the change in eGFR and the change in hemoglobin levels were included in the models as independent variables together with the baseline values (at age 70) and sex.

These results are given in Table C in S1 File. As could be seen, while the relationships between the changes in the proteins and the change in eGFR generally are inverse, the relationship vs change in hemoglobin levels could either be significantly positive (like for CASP-8), or negative (like for FGF-23), or insignificant (like for CX3CL1) with no obvious pattern found.

## Discussion

The present study demonstrates that plasma levels of a majority of the investigated cardiovascular-related proteins increased between age 70 and 80. We found an association between the change in levels of a great number of the proteins and a change in kidney function during the same period. Furthermore, changes in a number of the proteins were related to changes in hemoglobin levels, but in this case the relationships could either be positive or negative.

### Comparison with the literature

A couple of previous cross-sectional studies in adults have shown that many proteins are related with age, mainly in a positive fashion, i.e. higher age was associated with higher protein levels [2–5]. However, as far as we are aware, this is the first longitudinal study of a large number of proteins conducted in adults a crucial period of aging, providing novel and robust evidence for changes of proteins within adult individuals. Our findings in a fairly large adult cohort of an increase in the plasma levels in a majority of the investigated proteins support previous cross-sectional findings in adults.

Lietzen and co-workers described the longitudinal development of the proteome in 15 children from 0 to 3 years [6]. They found age to be a prominent factor influencing about half of the variation of the proteome. They found an increase in 35 proteins and a decline in 85 proteins over time. Liu and co-workers studied changes in the proteome in 10 healthy children from 9 month to 14 years [7]. Also in this study, about half of the evaluated proteins showed an age-related change, and of those, about half increased while the other half declined. Thus, the pattern of an increase in a majority of the proteins seen in our sample of elderly adults and in adult cross-sectional studies was not noted in these two longitudinal pediatric studies. It is likely that the changes in feeding pattern and growth development in children would influence the proteome in a different fashion as compared to adults.

Renal handling of proteins is not fully understood. In a study using untargeted proteomics of both plasma and urine, 2280 proteins were identified in plasma only, 1128 proteins were identified in urine only, while 394 proteins were found in both plasma and urine [19]. The proteins identified in plasma only generally had a larger theoretical molecular weight, consistent with the view that proteins with a high molecular weight are not filtered through the kidney [20], although some of those proteins could be actively secreted into the urine. The majority of the proteins found in both plasma and urine showed a theoretical molecular weight in the 15–45 kDa range, indicating that those proteins are filtered from plasma to the urine, but filtration is also influenced by the charge and shape of the protein.

Previous cross-sectional studies have shown that many proteins increase with declining GFR [8, 10]. Our finding of inverse associations between the change in plasma levels of many of the investigated proteins and the change in GFR gives further evidences for a link between a poor filtration in the kidneys and increased plasma protein levels. However, the causal mechanisms underlying this link are not clear. In the present study, we could not find any close relationship between the theoretical molecular weight and the change in protein level over the 10-year follow-up period. Furthermore, several of the proteins investigated in the present study have previously been shown to be associated with an accelerated future decline in GFR in a prospective study [21], implying that proteins might affect the future decline in GFR.

In addition, a reduction in GFR is associated with an altered tubular protein secretion, as well as synthesis of proteins, with reduced erythropoietin levels as a well-known example [22]. Thus, it is presently not clear if the inverse relationship between the change in plasma levels of many of the investigated proteins and the change in GFR mainly is due to a decline in filtration or other mechanisms like tubular secretion, or if high protein levels could affect GFR. To further address this uncertainty in causality, future studies are needed that simultaneously measure the proteome in plasma and urine together with GFR in a longitudinal fashion.

It should also be noted that several of the proteins that were most closely associated with changes in eGFR, like TNFR-1, TNFR-2 and TRAIL-R2, have been put forward as promising kidney damage biomarkers in previous studies [21, 23].

We found that the increase over time in several of the proteins were more pronounced in males than in females. However, since the decline in eGFR over time was not significantly different between males and females, a sex-specific decline in glomerular filtration is not likely to explain the sex-difference in increase in increase over time in several of the proteins.

We also hypothesized that a change in plasma water, as evaluated by a change in the hemoglobin value, would have an impact on the change in protein levels. Although we did notice that the change in hemoglobin levels were related to the change in many of the proteins, no clear pattern was found, as seen for eGFR. This might be to the fact that the hemoglobin value is affected by so many other factors then a change in plasma water, so that hemoglobin is not a valid marker of hemoconcentration, especially in the elderly with concomitant diseases.

### Strength and limitations

A major strength of the present study is the repeated measurements of a large number of proteins three times during a 10-year period, which allowed us to investigate the individual changes in those protein levels, as well as to relate them to changes kidney function and hemoglobin levels in a longitudinal fashion with a good statistical power. Using a longitudinal approach like ours is associated with less problems of reverse causality and confounding than the more commonly used cross-sectional design, although we acknowledge that causality still cannot be established using observational data. Our study also had several limitations. First, since we used a target proteomic chip designed with proteins known or suspected to be linked to cardiovascular disease, we cannot exclude that other proteins would display different longitudinal patterns and associations with examined variables. Second, since we studied individuals from Sweden, the generalizability to other ethnicities is unknown. Third, since we studied the change in proteins in the elderly, it cannot be taken for granted that the same results would appear in younger subjects. Fourth, only changes in proteins being linear over time were evaluated in the present study. However, with only three measurements performed, non-linear changes over could not be excluded, but with three measurements this is so far the most detailed examination of changes in multiple proteins over time.

In conclusion, the majority of evaluated proteins increased with aging. Therefore, normal ranges for proteins might be given in age-strata. This increase in protein levels was related to the degree of reduction in eGFR for the majority of proteins, while no clear pattern was seen for the relationships between the proteins and the change in hemoglobin levels.

## Supporting information

S1 File **Figure A.** Correlations of protein level changes over time. Only protein pairs with a correlation coefficient >0.60 are shown. **Figure B.** Associations between the change in hemoglobin level and change in 84 proteins over time (regression coefficient) and the–log_10_ p-value for these associations. The Bonferroni-corrected p-value is shown as the horizontal line. The regression coefficient (beta) gives the relationship between the change in protein levels (on a SD-scale) vs the change in hemoglobin level (in g/L). **Table A.** Basic characteristics in the sample at the three examinations. **Table B.** Beta, standard error (SE) and p-value for the interaction between time and sex regarding changes in 84 proteins over 10-year follow-up with measurements at ages 70, 75 and 80 years. A negative beta denotes that the change in the the protein over the ten years is more pronounced in men than in women. The direction of the changes in the proteins are given in Table 1 in the main manuscript. *P*< 0.00059 is regarded as significant (Bonferroni-correction for 84 proteins). **Table C.** Relationships between the change in the 84 proteins (dependent variable) and the changes in both glomerular filtration rate (GFR) and hemoglobin levels (both independent variables in the same model). The regression coefficient (beta) gives the relationship between the change in protein levels (on a SD-scale) vs the change in GFR (in ml/min/1.73m2) or the change in hemoglobin level (in g/L).
(DOCX)
