# Supplementary material for: Longitudinal effects of aging on plasma proteins levels in older adults – associations with kidney function and hemoglobin levels
Source: PLoS One. 2019 Feb 25;14(2):e0212060. doi: 10.1371/journal.pone.0212060 (PMC6388926; doi:10.1371/journal.pone.0212060)
Supplement: S1 File — Figure A. Correlations of protein level changes over time. Only protein pairs with a correlation coefficient >0.60 are shown. Figure B. Associations between the change in hemoglobin level and change in 84 proteins over time (regression coefficient) and the–log10 p-value for these associations. The Bonferroni-corrected p-value is shown as the horizontal line. The regression coefficient (beta) gives the relationship between the change in protein levels (on a SD-scale) vs the change in hemoglobin level (in g/L). Table A. Basic characteristics in the sample at the three examinations. Table B. Beta, standard error (SE) and p-value for the interaction between time and sex regarding changes in 84 proteins over 10-year follow-up with measurements at ages 70, 75 and 80 years. A negative beta denotes that the change in the the protein over the ten years is more pronounced in men than in women. The direction of the changes in the proteins are given in Table 1 in the main manuscript. P< 0.00059 is regarded as significant (Bonferroni-correction for 84 proteins). Table C. Relationships between the change in the 84 proteins (dependent variable) and the changes in both glomerular filtration rate (GFR) and hemoglobin levels (both independent variables in the same model). The regression coefficient (beta) gives the relationship between the change in protein levels (on a SD-scale) vs the change in GFR (in ml/min/1.73m2) or the change in hemoglobin level (in g/L). (DOCX) [file pone.0212060.s001.docx]

**Supplementary Figure A.** Correlations of protein level changes over time. Only protein pairs with a correlation coefficient >0.60 are shown.

**Supplementary Figure B.** Associations between the change in hemoglobin level and change in 84 proteins over time (regression coefficient) and the –log_10_ p-value for these associations. The Bonferroni-corrected p-value is shown as the horizontal line. The regression coefficient (beta) gives the relationship between the change in protein levels (on a SD-scale) vs the change in hemoglobin level (in g/L).

**Supplementary Table A.** Basic characteristics in the sample at the three examinations.

|  | Age 70 | Age 75 | Age 80 |
| --- | --- | --- | --- |
| Variable | Mean (SD) or proportion | Mean (SD) or proportion | Mean (SD) or proportion |
| Sex (% females) | 50 | 50 | 49 |
| Body mass index (kg/m^2^) | 27.0 (4.3) | 26.8 (4.3) | 26.9 (4.5) |
| Fasting glucose (mmol/l) | 5.3 (1.6) | 5.2 (1.4) | 5.2 (1.3) |
| Systolic blood pressure (mmHg) | 149 (22) | 148 (19) | 146 (19) |
| LDL-cholesterol (mmol/l) | 3.38 (0.88) | 3.37 (0.94) | 3.20 (0.90) |
| HDL-cholesterol (mmol/l) | 1.51 (0.43) | 1.49 (0.46) | 1.38 (0.39) |
| eGFR ( | 86.3 (16.3) | 70.8 (13.6) | 62.1 (14.6) |
| Hemoglobin | 137 (11) | 139 (11) | 136 (11) |
| Current smoker (%) | 11 | 6 | 3 |
| Myocardial infarction (%) | 7 | 9 | 11 |
| Stroke (%) | 4 | 7 | 10 |
| Diabetes (%) | 9 | 12 | 12 |

**Supplementary Table B.** Beta, standard error (SE) and p-value for the interaction between time and sex regarding changes in 84 proteins over 10-year follow-up with measurements at ages 70, 75 and 80 years. A negative beta denotes that the change in the the protein over the ten years is more pronounced in men than in women. The direction of the changes in the proteins are given in table 1 in the main manuscript. *P*< 0.00059 is regarded as significant (Bonferroni-correction for 84 proteins).

| **Protein** | **Beta** | **SE** | **p-value** |
| --- | --- | --- | --- |
| Matrix metalloproteinase-3 (MMP-3) | -.042 | .006 | 8.11e-12 |
| Leptin (LEP) | -.028 | .004 | 1.53e-10 |
| Growth hormone (GH) | -.041 | .008 | 1.10e-07 |
| N-terminal pro-B-type natriuretic peptide (NT-pro-BNP) | -.028 | .005 | 1.80e-07 |
| Follistatin (FS) | -.04 | .008 | 2.64e-07 |
| TNF-related apoptosis-inducing ligand receptor 2 (TRAIL-R2) | -.03 | .006 | 1.20e-06 |
| Agouti-related protein (AGRP) | .03 | .006 | 1.84e-06 |
| Prolactin (PRL) | -.027 | .007 | .0000396 |
| Cystatin-B (CSTB) | -.025 | .006 | .0000462 |
| Vascular endothelial growth factor D (VEGF-D) | -.021 | .005 | .0000615 |
| Tissue-type plasminogen activator (t-PA) | .023 | .006 | .000268 |
| Receptor for advanced glycosylation end products (RAGE) | -.02 | .006 | .000546 |
| Placenta growth factor (PlGF) | -.024 | .007 | .0007401 |
| Growth/differentiation factor 15 (GDF-15) | -.018 | .005 | .0007666 |
| Tumor necrosis factor receptor 1 (TNF-R1) | -.02 | .006 | .0016115 |
| Adrenomedullin (AM) | .018 | .006 | .0018092 |
| Galectin-3 (Gal-3) | -.018 | .006 | .0037962 |
| Monocyte chemotactic protein 1 (MCP-1) | -.021 | .007 | .0051708 |
| CD40L receptor (CD40) | -.017 | .006 | .0062225 |
| Matrix metalloproteinase-10 (MMP-10) | -.02 | .007 | .0065947 |
| Vascular endothelial growth factor A (VEGF-A) | -.017 | .006 | .0075134 |
| TNF-related activation-induced cytokine (TRANCE) | .017 | .007 | .0078579 |
| Tumor necrosis factor receptor 2 (TNF-R2) | -.017 | .006 | .0088765 |
| Matrix metalloproteinase-12 (MMP-12) | -.013 | .005 | .0088772 |
| Platelet-derived growth factor subunit B (PDGF subunit B) | .017 | .007 | .0121681 |
| Interleukin-1 receptor antagonist protein (IL-1RA) | -.015 | .006 | .0204449 |
| TNF-related apoptosis-inducing ligand (TRAIL) | .016 | .007 | .0210175 |
| Myeloperoxidase (MPO) | -.015 | .007 | .0213879 |
| Osteoprotegerin (OPG) | -.013 | .006 | .0230821 |
| C-X-C motif chemokine 16 (CXCL16) | -.017 | .008 | .025691 |
| Interleukin-8 (IL-8) | -.016 | .007 | .0268624 |
| Interleukin-16 (IL-16) | -.014 | .006 | .0268724 |
| E-selectin (SELE) | .011 | .005 | .032236 |
| Interleukin-27 subunit alpha (IL27-A) | -.011 | .005 | .0346176 |
| Dickkopf-related protein 1 (DKK-1) | .014 | .007 | .0451613 |
| C-X-C motif chemokine 1 (CXCL1) | .012 | .006 | .0451672 |
| Spondin-1 (SPON1) | -.014 | .007 | .0454618 |
| Urokinase plasminogen activator surface receptor (U-PAR) | -.011 | .006 | .0519035 |
| C-C motif chemokine 3 (CCL3) | -.012 | .006 | .0556397 |
| Fatty acid-binding protein 4 (FABP4) | .011 | .006 | .0561469 |
| P-selectin glycoprotein ligand 1 (PSGL-1) | -.015 | .008 | .0681821 |
| Fibroblast growth factor 23 (FGF-23) | .011 | .007 | .1024517 |
| Chitinase-3-like protein 1 (CHI3L1) | -.009 | .006 | .1067723 |
| C-X-C motif chemokine 6 (CXCL6) | .009 | .006 | .1117001 |
| Kallikrein-6 (KLK6) | -.01 | .007 | .1131131 |
| Macrophage colony-stimulating factor 1 (CSF-1) | -.011 | .007 | .1260122 |
| ST2 protein (ST2) | .008 | .006 | .1384986 |
| Kallikrein-11 (hK11) | -.009 | .006 | .1407591 |
| Membrane-bound aminopeptidase P (mAmP) | .005 | .004 | .1574398 |
| Resistin (RETN) | -.009 | .006 | .1598275 |
| Pentraxin-related protein PTX3 (PTX3) | -.009 | .007 | .1758453 |
| T-cell immunoglobulin and mucin domain 1 (TIM-1) | -.007 | .006 | .1889877 |
| Hepatocyte growth factor (HGF) | .008 | .006 | .2117226 |
| Caspase-8 (CASP-8) | .009 | .007 | .2410871 |
| Tumor necrosis factor ligand superfamily member 14 (TNFSF14) | .009 | .007 | .2431941 |
| Pappalysin-1 (PAPPA) | .006 | .006 | .2929861 |
| C-C motif chemokine 4 (CCL4) | .006 | .006 | .2945032 |
| Endothelial cell-specific molecule 1 (ESM-1) | -.006 | .006 | .3361235 |
| Matrix metalloproteinase-1 (MMP-1) | -.005 | .006 | .3625265 |
| Lectin-like oxidized LDL receptor 1 (LOX-1) | -.007 | .008 | .3813121 |
| Epidermal growth factor (EGF) | .007 | .008 | .413711 |
| Heat shock 27 kDa protein (HSP 27) | .007 | .009 | .4537893 |
| Myoglobin (MB) | .005 | .007 | .4565089 |
| Fractalkine (CX3CL1) | -.005 | .006 | .4578314 |
| Stem cell factor (SCF) | .004 | .006 | .4726748 |
| Eosinophil cationic protein (ECP) | -.004 | .006 | .4844126 |
| Proto-oncogene tyrosine-protein kinase Src (SRC) | .005 | .008 | .4856073 |
| Platelet endothelial cell adhesion molecule (PECAM-1) | -.004 | .007 | .5231415 |
| Interleukin-18 (IL-18) | -.003 | .005 | .5321532 |
| Interleukin-6 (IL-6) | -.004 | .007 | .5347054 |
| Cathepsin D (CTSD) | .004 | .006 | .5465154 |
| Galanin peptides (GAL) | -.003 | .005 | .5670736 |
| Ovarian cancer-related tumor marker CA 125 (CA-125) | .003 | .006 | .5723579 |
| Angiopoietin-1 receptor (TIE2) | .004 | .007 | .5744812 |
| Renin (REN) | -.002 | .006 | .6922125 |
| Protein S100-A12 (EN-RAGE) | -.004 | .009 | .6950411 |
| Interleukin-6 receptor subunit alpha (IL-6RA) | -.002 | .005 | .7632615 |
| Cathepsin L1 (CTSL1) | .003 | .009 | .7732623 |
| Proteinase-activated receptor 1 (PAR-1) | -.001 | .006 | .835812 |
| Thrombomodulin (TM) | -.001 | .007 | .8450567 |
| Tumor necrosis factor receptor superfamily member 6 (FAS) | -.001 | .007 | .8681627 |
| C-C motif chemokine 20 (CCL20) | -.001 | .007 | .9185919 |
| Heparin-binding EGF-like growth factor (HB-EGF) | .001 | .007 | .9239128 |
| Tissue factor (TF) | 0 | .007 | .9932604 |

**Supplementary Table C.** Relationships between the change in the 84 proteins (dependent variable) and the changes in both glomerular filtration rate (GFR) and hemoglobin levels (both independent variables in the same model). The regression coefficient (beta) gives the relationship between the change in protein levels (on a SD-scale) vs the change in GFR (in ml/min/1.73m2) or the change in hemoglobin level (in g/L).

|  | GFR | | | Hemoglobin | | |
| --- | --- | --- | --- | --- | --- | --- |
| Protein | beta | se | p-value | beta | se | p-value |
| Agouti-related protein (AGRP) | -.01 | .002 | 2.97e-08 | -.004 | .002 | .0537 |
| Adrenomedullin (AM) | -.004 | .002 | .0117692 | -.01 | .002 | 1.05e-07 |
| Caspase-8 (CASP-8) | -.019 | .002 | 4.74e-29 | .018 | .002 | 2.01e-19 |
| Ovarian cancer-related tumor marker CA 125 (CA-125) | -.006 | .002 | .0004827 | .002 | .002 | .4273073 |
| C-C motif chemokine 20 (CCL20) | -.008 | .002 | .0002026 | -.002 | .002 | .4454538 |
| C-C motif chemokine 3 (CCL3) | -.015 | .002 | 5.53e-16 | -.007 | .002 | .0005242 |
| C-C motif chemokine 4 (CCL4) | -.004 | .002 | .0073945 | .003 | .002 | .1458035 |
| CD40L receptor (CD40) | -.027 | .002 | 7.80e-61 | -.007 | .002 | .0005225 |
| Chitinase-3-like protein 1 (CHI3L1) | -.01 | .002 | 1.58e-08 | -.005 | .002 | .0080806 |
| Macrophage colony-stimulating factor 1 (CSF-1) | -.02 | .002 | 3.28e-25 | -.006 | .002 | .0121172 |
| Cystatin-B (CSTB) | -.027 | .002 | 3.80e-65 | -.004 | .002 | .0334223 |
| Cathepsin D (CTSD) | -.006 | .002 | .000128 | .008 | .002 | .0000563 |
| Cathepsin L1 (CTSL1) | -.018 | .002 | 2.91e-15 | .02 | .003 | 2.08e-14 |
| Fractalkine (CX3CL1) | -.018 | .002 | 3.53e-23 | .001 | .002 | .7536379 |
| C-X-C motif chemokine 16 (CXCL16) | -.015 | .002 | 1.49e-13 | -.004 | .002 | .1177161 |
| C-X-C motif chemokine 1 (CXCL1) | .001 | .002 | .5757366 | .007 | .002 | .0020673 |
| C-X-C motif chemokine 6 (CXCL6) | .001 | .002 | .8646792 | .013 | .002 | 2.66e-11 |
| Dickkopf-related protein 1 (DKK-1) | -.002 | .002 | .22742 | .012 | .002 | 2.42e-07 |
| Eosinophil cationic protein (ECP) | -.012 | .002 | 5.81e-12 | .01 | .002 | 1.20e-06 |
| Epidermal growth factor (EGF) | .002 | .002 | .4494225 | .001 | .003 | .6154218 |
| Protein S100-A12 (EN-RAGE) | -.018 | .002 | 1.99e-15 | .009 | .003 | .0010093 |
| Endothelial cell-specific molecule 1 (ESM-1) | -.005 | .002 | .0028584 | -.004 | .002 | .0773725 |
| Fatty acid-binding protein 4 (FABP4) | -.021 | .002 | 3.75e-38 | -.001 | .002 | .7719585 |
| Tumor necrosis factor receptor superfamily member 6 (FAS) | -.016 | .002 | 1.11e-16 | .004 | .002 | .0766812 |
| Fibroblast growth factor 23 (FGF-23) | -.025 | .002 | 3.20e-50 | -.014 | .002 | 5.84e-11 |
| Follistatin (FS) | -.008 | .002 | .0004597 | -.001 | .003 | .8280732 |
| Galanin peptides (GAL) | -.002 | .001 | .1257166 | .003 | .002 | .0443393 |
| Growth/differentiation factor 15 (GDF-15) | -.013 | .002 | 4.30e-18 | -.016 | .002 | 6.95e-19 |
| Growth hormone (GH) | .001 | .002 | .6854944 | -.009 | .003 | .0007027 |
| Galectin-3 (Gal-3) | -.016 | .002 | 2.27e-21 | .001 | .002 | .6201912 |
| Heparin-binding EGF-like growth factor (HB-EGF) | -.011 | .002 | 6.91e-08 | .002 | .002 | .5032245 |
| Hepatocyte growth factor (HGF) | -.013 | .002 | 3.61e-14 | .012 | .002 | 9.71e-10 |
| Heat shock 27 kDa protein (HSP 27) | -.006 | .002 | .0130717 | -.002 | .003 | .4721592 |
| Interleukin-27 subunit alpha (IL27-A) | -.003 | .001 | .0338426 | -.007 | .002 | .0001623 |
| Interleukin-16 (IL-16) | -.01 | .002 | 7.44e-09 | .001 | .002 | .8143601 |
| Interleukin-18 (IL-18) | -.005 | .002 | .0004858 | .005 | .002 | .0073319 |
| Interleukin-1 receptor antagonist protein (IL-1RA) | -.011 | .002 | 1.17e-08 | -.005 | .002 | .0181461 |
| Interleukin-6 receptor subunit alpha (IL-6RA) | -.011 | .002 | 3.45e-12 | .012 | .002 | 2.75e-11 |
| Interleukin-6 (IL-6) | -.011 | .002 | 3.82e-08 | -.008 | .002 | .0011889 |
| Interleukin-8 (IL-8) | -.006 | .002 | .0026276 | -.003 | .002 | .1856604 |
| Kallikrein-6 (KLK6) | -.014 | .002 | 4.75e-15 | -.007 | .002 | .0015394 |
| Leptin (LEP) | -.007 | .001 | 9.04e-09 | .004 | .002 | .0047829 |
| Lectin-like oxidized LDL receptor 1 (LOX-1) | -.016 | .002 | 2.14e-14 | .027 | .002 | 2.38e-28 |
| Myoglobin (MB) | -.02 | .002 | 7.73e-28 | .005 | .002 | .0151241 |
| Monocyte chemotactic protein 1 (MCP-1) | -.018 | .002 | 8.69e-19 | .001 | .002 | .8686147 |
| Matrix metalloproteinase-10 (MMP-10) | -.009 | .002 | 4.29e-06 | -.012 | .002 | 1.68e-06 |
| Matrix metalloproteinase-12 (MMP-12) | -.007 | .001 | 8.21e-06 | -.007 | .002 | .000052 |
| Matrix metalloproteinase-1 (MMP-1) | 0 | .002 | .9349576 | -.005 | .002 | .0064283 |
| Matrix metalloproteinase-3 (MMP-3) | -.007 | .002 | .0000372 | -.01 | .002 | 1.02e-06 |
| Myeloperoxidase (MPO) | -.011 | .002 | 1.61e-09 | .007 | .002 | .0014442 |
| N-terminal pro-B-type natriuretic peptide (NT-pro-BNP) | -.005 | .002 | .0016237 | -.013 | .002 | 1.98e-13 |
| Osteoprotegerin (OPG) | -.009 | .002 | 1.63e-07 | .005 | .002 | .0090877 |
| Pappalysin-1 (PAPPA) | .004 | .002 | .0117304 | .004 | .002 | .0258971 |
| Proteinase-activated receptor 1 (PAR-1) | -.014 | .002 | 1.01e-17 | -.01 | .002 | 9.40e-08 |
| Platelet-derived growth factor subunit B (PDGF subunit B) | .001 | .002 | .6502385 | .018 | .002 | 2.19e-15 |
| Platelet endothelial cell adhesion molecule (PECAM-1) | -.009 | .002 | 2.17e-06 | .015 | .002 | 2.38e-11 |
| Prolactin (PRL) | -.01 | .002 | 2.12e-07 | -.006 | .002 | .0064523 |
| P-selectin glycoprotein ligand 1 (PSGL-1) | -.009 | .002 | .0000169 | .009 | .003 | .0009121 |
| Pentraxin-related protein PTX3 (PTX3) | -.008 | .002 | .000012 | .002 | .002 | .2768472 |
| Placenta growth factor (PlGF) | -.023 | .002 | 5.59e-35 | -.005 | .002 | .0448137 |
| Receptor for advanced glycosylation end products (RAGE) | -.013 | .002 | 9.43e-14 | -.003 | .002 | .1203673 |
| Renin (REN) | -.024 | .002 | 2.01e-46 | -.002 | .002 | .2294265 |
| Resistin (RETN) | -.012 | .002 | 3.33e-11 | -.004 | .002 | .0539753 |
| Stem cell factor (SCF) | -.007 | .002 | .0000729 | .007 | .002 | .0004948 |
| E-selectin (SELE) | -.007 | .002 | 4.73e-06 | .013 | .002 | 9.67e-14 |
| Spondin-1 (SPON1) | -.014 | .002 | 1.48e-14 | .003 | .002 | .2518561 |
| Proto-oncogene tyrosine-protein kinase Src (SRC) | .001 | .002 | .889757 | -.005 | .002 | .0400455 |
| ST2 protein (ST2) | -.008 | .002 | 9.89e-07 | .001 | .002 | .5309818 |
| Tissue factor (TF) | -.015 | .002 | 2.44e-15 | .003 | .002 | .1317627 |
| Angiopoietin-1 receptor (TIE2) | -.009 | .002 | .0000132 | .018 | .002 | 7.52e-14 |
| T-cell immunoglobulin and mucin domain 1 (TIM-1) | -.016 | .002 | 1.67e-22 | -.005 | .002 | .0149069 |
| Thrombomodulin (TM) | -.015 | .002 | 2.14e-15 | -.001 | .002 | .7136707 |
| Tumor necrosis factor ligand superfamily member 14 (TNFSF14) | -.01 | .002 | 4.82e-07 | .009 | .002 | .0001504 |
| Tumor necrosis factor receptor 1 (TNF-R1) | -.028 | .002 | 3.30e-73 | -.012 | .002 | 8.86e-10 |
| Tumor necrosis factor receptor 2 (TNF-R2) | -.027 | .002 | 1.80e-61 | -.008 | .002 | .0001276 |
| TNF-related apoptosis-inducing ligand receptor 2 (TRAIL-R2) | -.026 | .002 | 9.3e-62 | -.008 | .002 | .0000463 |
| TNF-related apoptosis-inducing ligand (TRAIL) | .001 | .002 | .496943 | .007 | .002 | .003986 |
| TNF-related activation-induced cytokine (TRANCE) | .001 | .002 | .924068 | .007 | .002 | .0029972 |
| Urokinase plasminogen activator surface receptor (U-PAR) | -.012 | .002 | 2.11e-15 | -.009 | .002 | 3.65e-07 |
| Vascular endothelial growth factor A (VEGF-A) | -.016 | .002 | 4.16e-18 | -.01 | .002 | 1.59e-06 |
| Vascular endothelial growth factor D (VEGF-D) | .001 | .002 | .8643003 | -.002 | .002 | .2086854 |
| Kallikrein-11 (hK11) | -.019 | .002 | 4.30e-29 | -.004 | .002 | .0272076 |
| Membrane-bound aminopeptidase P (mAmP) | .004 | .001 | .0009408 | .006 | .001 | 6.87e-07 |
| Tissue-type plasminogen activator (t-PA) | -.006 | .002 | .0006694 | .014 | .002 | 7.87e-11 |
